# Human Mesenchymal Stem Cells as a Carrier for a Cell-Mediated Drug Delivery

**DOI:** 10.3389/fbioe.2022.796111

**Published:** 2022-02-24

**Authors:** L. S. Litvinova, V. V. Shupletsova, O. G. Khaziakhmatova, A. G. Daminova, V. L. Kudryavtseva, K. A. Yurova, V. V. Malashchenko, N. M. Todosenko, V. Popova, R. I. Litvinov, E. I. Korotkova, G. B. Sukhorukov, A. J. Gow, D. Weissman, E. N. Atochina-Vasserman, I. A. Khlusov

**Affiliations:** ^1^ Center for Immunology and Cellular Biotechnology, Immanuel Kant Baltic Federal University, Kaliningrad, Russia; ^2^ Institute of Fundamental Medicine and Biology, Kazan Federal University, Kazan, Russia; ^3^ Kazan Institute of Biochemistry and Biophysics, FRC KSC of RAS, Kazan, Russia; ^4^ Interdisciplinary Center for Analytical Microscopy, Kazan Federal University, Kazan, Russia; ^5^ School of Engineering and Materials Science, Queen Mary University of London, London, United Kingdom; ^6^ School of Earth Sciences and Engineering, National Research Tomsk Polytechnic University, Tomsk, Russia; ^7^ Department of Medicine, Perelman School of Medicine, University of Pennsylvania, Philadelphia, PA, United States; ^8^ Skolkovo Institute of Science and Technology, Moscow, Russia; ^9^ Department of Pharmacology and Toxicology, Ernest Mario School of Pharmacy, Rutgers, The State University of New Jersey, Piscataway, NJ, United States; ^10^ Department of Morphology and General Pathology, Siberian State Medical University, Tomsk, Russia; ^11^ Research School of Chemistry and Applied Biomedical Sciences, National Research Tomsk Polytechnic University, Tomsk, Russia

**Keywords:** human adipose-derived mesenchymal stromal/stem cells, cell migration, target drug delivery, cell-mediated drug delivery, synthetic microcapsules

## Abstract

A number of preclinical and clinical studies have demonstrated the efficiency of mesenchymal stromal cells to serve as an excellent base for a cell-mediated drug delivery system. Cell-based targeted drug delivery has received much attention as a system to facilitate the uptake a nd transfer of active substances to specific organs and tissues with high efficiency. Human mesenchymal stem cells (MSCs) are attracting increased interest as a promising tool for cell-based therapy due to their high proliferative capacity, multi-potency, and anti-inflammatory and immunomodulatory properties. In particular, these cells are potentially suitable for use as encapsulated drug transporters to sites of inflammation. Here, we studied the *in vitro* effects of incorporating synthetic polymer microcapsules at various microcapsule-to-cell ratios on the morphology, ultrastructure, cytokine profile, and migration ability of human adipose-derived MSCs at various time points post-phagocytosis. The data show that under appropriate conditions, human MSCs can be efficiently loaded with synthesized microcapsules without damaging the cell’s structural integrity with unexpressed cytokine secretion, retained motility, and ability to migrate through 8 μm pores. Thus, the strategy of using human MSCs as a delivery vehicle for transferring microcapsules, containing bioactive material, across the tissue–blood or tumor–blood barriers to facilitate the treatment of stroke, cancer, or inflammatory diseases may open a new therapeutic perspective.

## Introduction

Messenger RNA (mRNA)- and DNA-based therapies are a rapidly developing direction in the biomedical field. However, efficient delivery of genetic material and other bioactive molecules, such as proteins and small molecules, to specific target sites remains a challenge. Lipid nanoparticles (LNPs) are the most clinically advanced non-viral gene delivery system because of their high penetrating ability, biocompatibility, and delivery efficiency (Buschmann et al., 2021; Hou et al., 2021; Limongi et al., 2021). LNPs are widely used for the delivery of nucleic acids because of their efficacy in delivery along with their simple synthesis, small size, and serum stability (Mitchell et al., 2021). Ionizable LNP and ionizable amphiphilic Janus dendrimer (IAJD) delivery systems for mRNA are an ideal platform for the delivery of these nucleic acid therapies (Zhang et al., 2021). However, despite many advantages, LNP and IAJD systems are incapable of delivering formulated active substances to designated organs and tissues other than the liver and spleen with high efficiency (Fenton et al., 2018). Thus, there is an urgent need for a delivery system that is similar to LNP in terms of biocompatibility, cell uptake, and release, but is also capable of transferring genetic material with its long-term protection to the vast majority of specific target sites.

Polymeric, hybrid micron and submicron-sized multilayer capsules are a developing universal platform for safe encapsulation and efficient delivery of biomacromolecules (Petrov et al., 2005; Timin et al., 2018b). The significant advantages of these microcapsules as a delivery carrier for genetic material compared with other non-viral carriers include their superior loading capacity, low cytotoxicity, and high colloidal stability (Lepik et al., 2016). In addition, microcapsules are readily internalized inside a cell without evident alterations to the carrier cell function (Lepik et al., 2016), metabolic activity (Zelikin et al., 2010), or changing the biological activity of the encapsulated drug (Timin et al., 2018b). Cell functionalization with recently developed nano- and microcarriers for therapeutics has significantly expanded the application of cell therapy and targeted drug delivery (Timin et al., 2018a). One potential viable approach is the creation of cell-based delivery systems enabling the uptake and transfer of active substances to designated organs and tissues with high efficiency.

Human mesenchymal stem cells or multipotent mesenchymal stromal cells (hMSCs) are undifferentiated cells, possessing the capability of self-renewal and differentiation into various mesenchymal tissues, most prominently bone, cartilage, and adipose. According to the International Society for Cellular Therapy, plastic adherent cells that differentiate into osteoblasts, adipocytes, and chondroblasts *in vitro*, expressing CD105, CD90, CD73, and CD44, and are characterized by a lack of CD45, CD34, CD14, CD11b, CD79, CD19, and HLA-DR expression can be considered human multipotent mesenchymal stromal cells (Dominici et al., 2006). Mesenchymal stem cells were first isolated from the bone marrow in 1968 (Friedenstein et al., 1968), and since then, they have been isolated from a variety of other tissues, including adipose (Zuk et al., 2002), perivasculature (Crisan et al., 2008), dental pulp (Gronthos et al., 2000), muscle, dermis (Young et al., 2001), and fetal tissue (Campagnoli et al., 2001; Wang et al., 2004). Bone marrow–derived MSCs and adipose-derived MSCs are commonly used in regenerative medicine (Gamie et al., 2012; Bajek et al., 2016). MSCs fulfill the general accepted criteria for cell-based therapies, but still need further investigation into their efficiency (Mazini et al., 2020). It has been reported that hMSCs loaded with a drug can deliver therapeutic cytokines to injury or inflammatory sites, thus finding application in regeneration (Huang et al., 2012) and antitumor therapy (Hu et al., 2010). One of the key features of hMSC-based therapies is their ability to migrate toward injury and to preferentially hone to damaged tissue. Thus, hMSCs are attractive candidates as cargo containers for carrying therapeutic drugs and bioactive materials to specific target sites. The major problem that arises is how to incorporate a foreign active substance into cells and maintain their stability in the carrier until delivery to the targeted sites triggers release.

Herein, we investigated a new cell-based drug delivery system based on human MSCs and their direct interactions with synthetic polymer microcapsules (as potential “cargo containers”), their biocompatibility, and loading efficiency (Zyuzin et al., 2019). We determined optimized time- and dose-dependent conditions for the most efficient internalization of microcapsules by hMSCs with minimal cytotoxicity as a precursor to develop a drug-delivery system. We have shown the ability of hMSCs to efficiently uptake the microcapsules over time at different cell to capsule ratios and determined the ratios of microcapsules that have a negligible effect on cell morphology, ultrastructure, motility, and migration activity. Taken together, this work suggests that human MSCs have sufficiently high biocompatibility with synthetic microcapsules to be used as drug delivery carriers in future studies.

## Materials and Methods

### Materials

Bovine serum albumin (BSA, MW), fluorescein isothiocyanate isomer I (FITC), phosphate-buffered saline (PBS), calcium chloride, sodium carbonate, poly (allylamine hydrochloride) (PAH), poly (sodium 4-styrenesulfonate) (PSS), minimum Essential Medium Eagle Alpha Modification (α-MEM), ITS Liquid Media Supplement, fetal bovine serum (FBS), L-glutamine, and penicillin/streptomycin were purchased from Sigma–Aldrich (St. Louis, MO, United States). Ethylenediaminetetraacetic acid (EDTA) was purchased from Helicon, MSC Phenotyping Kit (Miltenyi Biotec, Bergisch Gladbach, Germany), and trypan blue solution (Invitrogen, United Kingdom).

### Isolation and Culturing Human MSCs

Human mesenchymal stem cells were isolated from lipoaspirates of healthy donors with signed informed consent according to the guidelines approved by the Local Ethics Committee of Innovation Park, Immanuel Kant Baltic Federal University, Kaliningrad, Russia (permission no. 7 on 9 December 2015). Informed consent for the procedure was obtained as specified previously (Avdeeva et al., 2019). A stromal vascular fraction and a processed lipoaspirate (PLA), containing a small fraction of endothelial cells, pericytes, and smooth muscle cells, were obtained as described elsewhere (Zuk et al., 2001). PLA was passaged at sub-confluence four times (each passage lasting 5–7 days) and cultured at 37°C and 5% CO_2_ in a nutrient medium consisting of 90% α-MEM (Sigma–Aldrich, St. Louis, MO, United States), 10% inactivated FBS (Sigma, United States), 0.3 g/L L-glutamine (Sigma, United States), and 100 U/ml penicillin/streptomycin (Sigma–Aldrich, United States) to expand the *ex vivo* human adipose-derived stromal/stem cell population. The hMSCs were detached from the plastic wells with 0.05% trypsin (PanEco, Russia) in 0.53 mM EDTA (Sigma–Aldrich, St. Louis, MO, United States) and washed twice with PBS. Evaluation of the expression of the CD14, CD20, CD34, CD45, CD73, CD90, and CD105 surface markers of the viable hMSCs was performed using a MSC Phenotyping Kit according to the manufacturer’s protocol (Miltenyi Biotec, Bergisch Gladbach, Germany) and Viability Fixable Dyes (Miltenyi Biotec, Bergisch Gladbach, Germany). Following a 10-min incubation with the labeled mAbs [fluorescein isothiocyanate (FITC), allophycocyanin (APC), phycoerythrin (PE), or peridinin chlorophyll protein (PerCP)], the cells were analyzed by flow cytometry with a MACS Quant flow cytometer (Miltenyi Biotec, Bergisch-Gladbach, Germany) and KALUZA Analysis Software (Beckman Coulter, Brea, CA, United States) in accordance with the manufacturer’s instructions. The concentration and viability of cells were determined with a Countess™ Automated Cell Counter (Invitrogen, United Kingdom) using 0.4% trypan blue solution (Invitrogen, United Kingdom). Viable cells (93–95%) exhibited high expression of CD73 (94–99%), CD90 (87–98%), and CD105 (77–98%) antigens, and a very low expression (1.4–1.9%) of hematopoietic immunophenotype markers (CD45, CD34, CD20, and CD14). After 21-day culturing in specific induction media (StemPro^®^ Differentiation Kit, Thermo Fisher Scientific, Waltham, MA, United States) fibroblast-like adherent adipose-derived stromal/stem cells showed multilineage differentiation into osteoblasts, chondrocytes, and adipocytes by selective staining as previously described (Litvinova et al., 2018; Avdeeva et al., 2019; Khlusov et al., 2020). Thus, the isolated cells constitute a pool of adipose-derived hMSCs according to the definitions of the International Society for Cellular Therapy and the International Federation for Adipose Therapeutics and Science (IFATS) (Dominici et al., 2006; Bourin et al., 2013).

### Microcapsule Synthesis and Characterization

Microcapsules were synthesized using a layer-by-layer method as previously described (Petrov et al., 2005). Briefly, microcapsules were prepared in a clean environment under sterile condition by using sacrificial porous vaterite (CaCO_3_) spherical particles (Supplementary Figure S1A) as a template for further polyelectrolyte absorption and capsule formation. To prepare vaterite spherical particle solutions of Na_2_CO_3_ (0.33 M) and CaCl_2_ (0.33 M), 2 ml of each were mixed and stirred for 30s at RT with a magnetic stirrer. When the process was finished, CaCO_3_ particles were washed thrice with deionized water. After that, PSS/PAH were consistently absorbed onto vaterite particles, resulting in three polyelectrolyte bilayers. Multilayer film on top of CaCO_3_ templates formed a polyelectrolyte multilayer with a thickness of several nanometers. BSA conjugated with fluorescein isothiocyanate isomer I (FITC-BSA) was used for the labeling of capsules for visualization. CaCO_3_ particles were dissolved with 0.2 M EDTA solution flowing by a triple wash with deionized water resulting in polyelectrolyte capsules with mean diameter 2.7 ± 0.2 µm. The number of capsules was determined with a hemocytometer.

Scanning electron microscopy (SEM, ESEM Quanta 400 FEG, FEI, United States) was used to investigate the morphology of obtained CaCO_3_ particles and layer-by-layer capsules with imaging conditions of 10 kV accelerating voltage and 10 mm working distance. Characteristic CaCO_3_ particles and capsules templated on these particles are shown in the Supplementary Material (Supplementary Figure S1). Vaterite core leaves a capsule after dissolving with distinct hollow inner cavity and gives the capsule a spherical shape (Supplementary Figure S1B) as described previously (Volodkin et al., 2004). Prior to the investigation, the material surface was coated with a thin gold layer (Agar Auto Sputter Coater, Agar Scientific, United Kingdom). The choice of synthetic polymers PSS and PAH for capsule fabrication was due to their stability for weeks while internalized by various biological cells, practicality in visualization, and lack of known cellular toxicity (Zyuzin et al., 2019).

### Characterization of hMSC Phenotypic Profile in Response to Cellular Loading

The obtained hMSCs were incubated with FITC-labeled synthetic microcapsules at various cell to microcapsule ratios (1:0, 1:5, 1:10, 1:20, 1:45, or 1:90) for 24 h at 37°C, 5% CO_2_ in the nutrient medium as described in the Methods; then the cells were washed with PBS to remove free microcapsules and further cultured for 6, 10, 24, 48, and 72 h. Cells were enumerated and analyzed for viability with 0.4% trypan blue solution and expression of surface markers (CD14, CD20, CD34, CD45, CD73, CD90, and CD105) by flow cytometry at 24 h post-phagocytosis as described above. All experiments were performed in triplicate. Cultured hMSCs incubated without internalized microcapsules were used as control. Microcapsule internalization was analyzed using the Cell-IQ v2 MLF integrated platform (CM Technologies Oy, Tampere, Finland) for continuous real-time phase-contrast imaging or by transmission electron microscopy.

### Cytokine Profile of Cultured hMSCs Loaded With Microcapsules

Cell culture media was collected at 24 and 48 h post-phagocytosis of various ratios of microcapsules. Collected supernatants were centrifuged at 500 g for 10 min and analyzed by fluorescence flow cytometry by measuring the spontaneous and microcapsule-induced secretion of the following human cytokines and chemokines: LIF, SCF, SDF-1a, SCGF-β, M-CSF, MCP-3, MIF, MIG, TRAIL, Gro-α; IL-1a, IL-2ra, IL-3, IL-12 (p40), IL-16, IL-18, HGF, TNF-β, β-NGF, IFN-α2, and CTACK. Fluorescence flow cytometry was conducted with monoclonal antibodies according to the manufacturer’s instructions for the cytokine assay system (Bio-Plex Pro Human Cytokine Group II 21-Plex Panel, Bio-Rad, Hercules, CA, United States) using an automated processing system (Bio-Plex Protein Assay System, Bio-Rad, Hercules, CA, United States). The concentration of each cytokine was presented in pg/ml, with *n* = 3–9 per group.

### Nitrite Measurements

Cell culture supernatants were collected at 24, 48, and 72 h post-phagocytosis with various ratios of microcapsules and analyzed for nitrite content *via* cyclic voltammetry. Prior to the assay, all proteins within the collected supernatant media were precipitated by the addition of concentrated trichloroacetic acid followed by centrifugation for 10 min, 14,000 rpm. Each sample was measured 3–5 times, with *n* = 3 per each time point. A standard curve for nitrite detection in cell media was *R* ^2^ = 0.99. Linear sweep voltammetry analyses were performed by using an end graphite indicator electrode and an auxiliary Ag/AgCl reference electrode. Brinton–Robinson buffer solution, pH 4.02 was used as a background electrolyte. The sweep speed potential is W = 100 mV/s, the operating range of potentials is from 0.4 to 1.5 V, the accumulation time of the substance on the electrode is 4 s, and the accumulation potential is 0.4 V.

### Transmission Electron Microscopy

The suspensions of hMSCs cultured with various ratios of microcapsules were fixed in 2.5% glutaraldehyde in Tyrode’s buffer for 1.5 h at room temperature and then centrifuged at 1500 g for 5 min. The precipitates were washed with Tyrode’s buffer and then postfixed with 1% osmium tetroxide in the same buffer supplemented with sucrose (25 mg/ml) for 2 h. The samples were dehydrated in ascending ethanol concentrations, acetone, and propylene oxide. The epoxy resin medium was Epon 812 (Fluka, Switzerland). The samples were allowed to polymerize for 3 days at increasing temperature from 37°C to 60°C. Ultrathin sections were cut using LKB ultramicrotome-III (LKB, Sweden) and stained with saturated aqueous uranyl acetate (Serva, Germany) at 60°C for 10 min and lead citrate (Serva, Germany) at room temperature for 5 min. The specimens were examined using an electron microscope, Jem-1200 EX (Jeol, Japan), at an operating voltage of 80 kV. The ultrastructural changes at each experimental condition studied were based on three independent hMSC preparations with at least three samples processed from each cell preparation. Not less than 40–50 ultrathin sections were viewed and analyzed followed by imaging of at least 150–200 cells at each experimental condition. Transmission electron microscopy was carried out on the equipment of CSF-SAC FRC KSC RAS (Kazan, Russia).

### Effect of Internalized Microcapsules on Cell Motility

The mobility of the cells with and without internalized microcapsules was monitored with a Cell-IQ^®^ v2 MLF integrated platform (CM Technologies Oy, Tampere, Finland) for continuous fluorescent and phase-contrast visualization of living cells in real time in accordance with the manufacturer’s instructions. Individual cells were marked by colored tags, and their images were traced for 24 h post-phagocytosis. Obtained hMSCs (50,000 viable cells per well, fifth passage) were incubated with FITC-labeled capsules at various ratios (1:0, 1:10, 1:20, 1:45, and 1:90 microcapsules per cell) for 24 h at 37°C, 5% CO_2_; then the cells were washed with PBS, collected, and transferred to the Cell-IQ system (37°C, 100% humidity and 5% СО_2_), and further cultured for an additional 24 h. To evaluate mobility of hMSCs with and without microcapsules, cells’ images were captured every 45 min, and 1,308 images were obtained for each of the indicated time points. Every 10^th^ image was used for automatic analysis with the Cell-IQ Imagen software. The maximum cell number and the time of cell division-initiation were determined in the chosen visualization points. At 6, 10, and 24 h post-phagocytosis, the cells were analyzed using the Cell-IQ device for a continuous real-time fluorescent and phase-contrast imaging.

### Evaluation of the Ability of hMSCs Loaded With Microcapsules to Migrate Through the 8-µm Pores

The ability of hMSCs loaded with microcapsules to migrate through 8 µm micropores in a polymer membrane was monitored with special 16-well CIM plates of the xCELLigence RTCA DP system (Roche Applied Science, Pennsburg, Germany). The system enabled a real-time determination of the impedance dynamics at the points of contact between the cells and the gold electrodes and of the cell index that pertained to the count and area of the cells attached to the electrodes. Each well in the CIM plate is composed of two chambers. The lower chamber contains chemoattractant, cells, or tissues, and test cells are placed in the upper chamber. According to the manufacturer’s recommendation, 2% FBS and 1% ITS (Sigma-Aldrich, United States) were added to α-MEM (Sigma–Aldrich, St. Louis, MO, United States) to eliminate the formation of bubbles when the CIM plate was assembled. The test was performed as described previously (Khlusov et al., 2020) with some modifications. Cells (40,000 per conditions) were incubated with FITC-labeled microcapsules at various ratios (1:0, 1:10, 1:20, and 1:45 microcapsules per cell) for 24 h at 37°C, 5% CO_2_; then cells were washed with PBS, collected, placed in the upper chamber, and allowed to migrate through 8 μm pores to the lower chamber (the opposite surface of the membrane in the upper chamber), which is 80% covered with gold electrodes (Litvinova et al., 2018). Four wells were used for each experimental group. Signals for the determination of the migration index for each well were registered with RTCA Software every 15 min up to 70 h.

### Statistics

The results were analyzed, and the Statistical analyses were conducted using the STATISTICA 13.3 software package for Windows 10.0 (TIBCO Software Inc., Palo Alto, CA, United States). Testing for normality was performed using the Shapiro–Wilk test. The mean (M) and standard deviation (SD) or median (Me), 25% (Q1), and 75% (Q3) quartiles were calculated. The non-parametric Mann–Whitney *U*-test and Wilcoxon test were used to determine the statistical significance of differences. The differences were statistically significant at *p* < .05.

## Results

### hMSC Phenotypic Activation in Response to Cellular Loading

**FIGURE 1:**
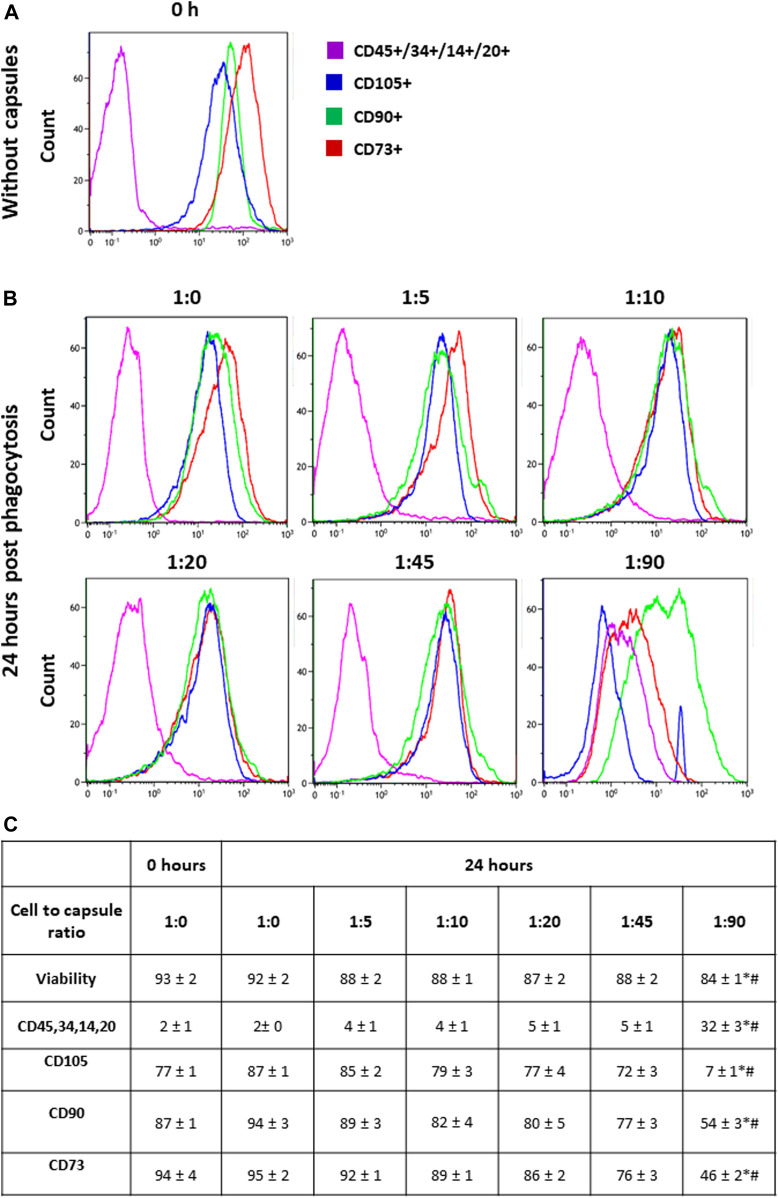
Phenotypic switching in response to cellular loading. Following capsule phagocytosis at ratios of 1:0, 1:5, 1:10, 1:20, 1:45, and 1:90 cells were incubated for additional 24 h and then analyzed for the expression of surface markers by flow cytometry. Cells were gated on viability, forward, and side scatter profile. **Panel (A)** shows the representative histogram of markers expression in hMSCs without capsules (baseline). **Panel (B)** shows the representative histogram of markers expression in hMSCs at 24 h post-phagocytosis at a cell/microcapsule ratio of 1:1, 1:5, 1:10, 1:20, 1:45, and 1:90. **Panel (C)** shows the data from three independent experiments. For each marker, the mean fluorescence intensity was calculated and averaged across the three experiments. The values in the table are averaged mean fluorescence intensity across the three experiments, mean + SD. *p < .05 comparison with 0 h; ^#^ *p* < .05 comparison with 24 h without microcapsules.

Human MSCs were isolated and cultured as described in the Methods and then analyzed by flow cytometry for the expression of surface markers. Figure 1A shows that the majority of cultured hMSCs in untreated condition express CD73, CD105, and CD90, while less than 10% of the cells express hematopoietic markers CD45, CD34, CD14, and CD20. Therefore, these cells retained the typical hMSC markers during long-term culturing on plastic in the standard medium. Figure 1C shows that hMSCs incubated with FITC-labeled microcapsules at ratios of 1:0, 1:5, 1:10, and 1:20 did not change their phenotype profile; however, incubation of cells at a microcapsule ratio of 1:45 caused a small but significant loss of all three main markers, namely, CD90, CD73, and CD105, but no increase in CD45, CD34, CD14, and CD20. In contrast, at a ratio of 1:90, CD90 and CD73 expression are decreased to 50% of total, and CD105 is reduced to less than 10%, while CD45, CD34, CD14, and CD20 expression were increased. These data indicate that at higher loads of microcapsules, internalization not only reduces cell viability but also switches phenotype toward that of a myeloid lineage.

To evaluate whether microcapsule internalization induces inflammatory activation of hMSCs, inflammatory cytokines and chemokines levels were analyzed in the intercellular media at 24 and 48 h post-phagocytosis of various ratios of microcapsules (Supplementary Table S1). These data showed that the majority of cytokines and chemokines were not significantly altered when hMSCs were loaded with microcapsules at 1:5, 1:20, and 1:45 ratios when compared with control (cells without capsules, 1:0 ratio), especially after 24-h culturing. There was a small but statistically significant alteration in cytokines and chemokine levels depending on microcapsule ratios at 48 h time point post-phagocytosis. However, all these changes are not relevant to the MSC since the secreted concentration is very low (1–100 pg/ml) according to van den Broek classification (Van den Broek et al., 2014). Thus, at lower ratios, there is minimal risk of “cytokine storm,” which is consistent with the cells maintaining their stem-like nature.

**FIGURE 2:**
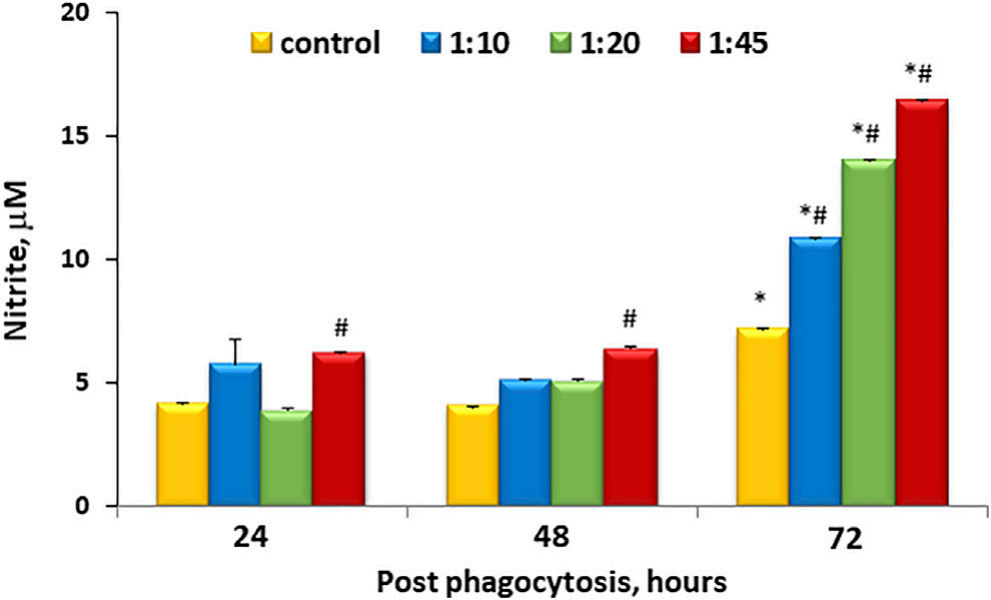
Dose-dependent microcapsule loading increases nitrite levels and cell activation. Cells were incubated with microcapsules at various ratios (1:0, 1:10, 1:20, and 1:45) and cultured for 24, 48, or 72 h post-phagocytosis; at the indicated time points, the supernatant media were collected and analyzed for nitrite content *via* cyclic voltammetry. Each sample was measured 3–5 times, *n* = 3 per each conditions and time points. *р < .05 *versus* the corresponding cell/microcapsule ratio at 24 h post phagocytosis; ^#^ *p* < .05 *versus* control at the same time point.

One of the earliest indicators of inflammatory activation is the production of NO as a result of nitric oxide synthase induction. This can be readily monitored by the measurement of accumulated nitrite in the medium. Figure 2 shows that there is accumulation of nitrite within the media over time even under control conditions. However, this accumulation was similar to control at ratios less than and equal to 1:20 at 48 h, but at 72 h, microcapsules at all ratios tested demonstrated higher levels of NO accumulation that were untreated.

**FIGURE 3:**
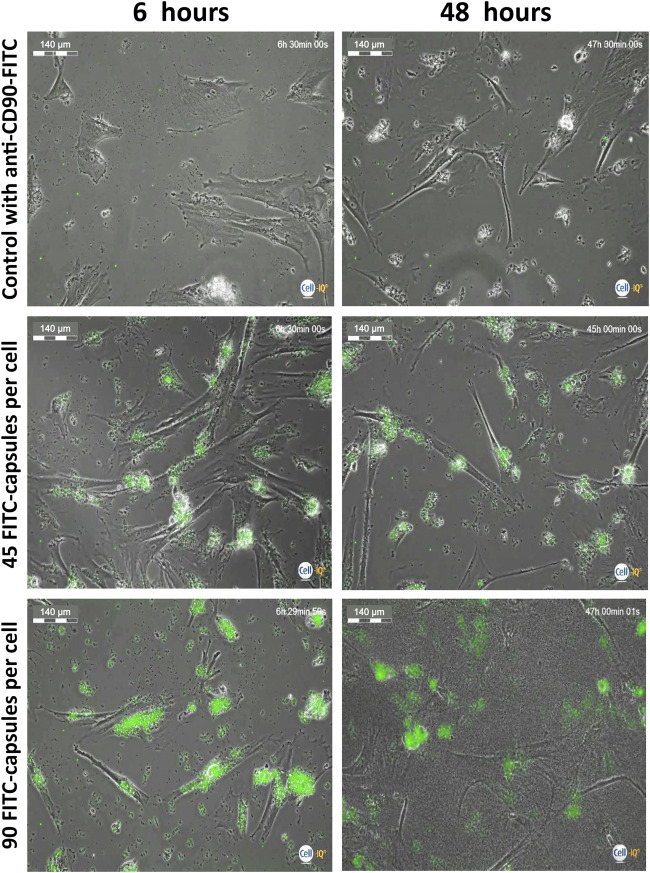
Evaluation of microcapsule internalization on cell morphology. Isolated human MSCs were incubated with FITC-labeled capsules at a ratio of 1:0 (control), 1:45, and 1:90 microcapsules per cell for 24 h at 37°C, then the cells were washed with PBS, collected, transferred to another plate, and further cultured for up to 48 h. At 6 and 48 h post-phagocytosis, cells were analyzed using the Cell-IQ v2MLF integrated platform for a continuous real-time phase-contrast imaging as described in the Methods. There is minimal change in cell morphology or death rate when capsules are used at a 1:45 ratio, relative to control, although significant microcapsule uptake is observed. However, at a ratio of 1:90, cell shrinkage is apparent. Scale bar is 140 microns.

To evaluate what appropriate time points should be used to study loaded cells’ motility, hMSCs were stressed with high doses of microcapsules and were analyzed at 6 and 48 h post-phagocytosis using the Cell-IQ v2MLF integrated platform, continuous real-time phase-contrast imaging. Figure 3 shows that there is minimal change in the cell morphology when microcapsules are used at a 1:45 ratio, relative to control, although significant capsule uptake is observed. However, at a ratio of 1:90, cell shrinkage is apparent. Thus, for further evaluation, we chose to use a ratio less than 1:45 and time less than 48 h.

### Stepwise Internalization of Synthetic Microcapsules by hMSCs

**FIGURE 4:**
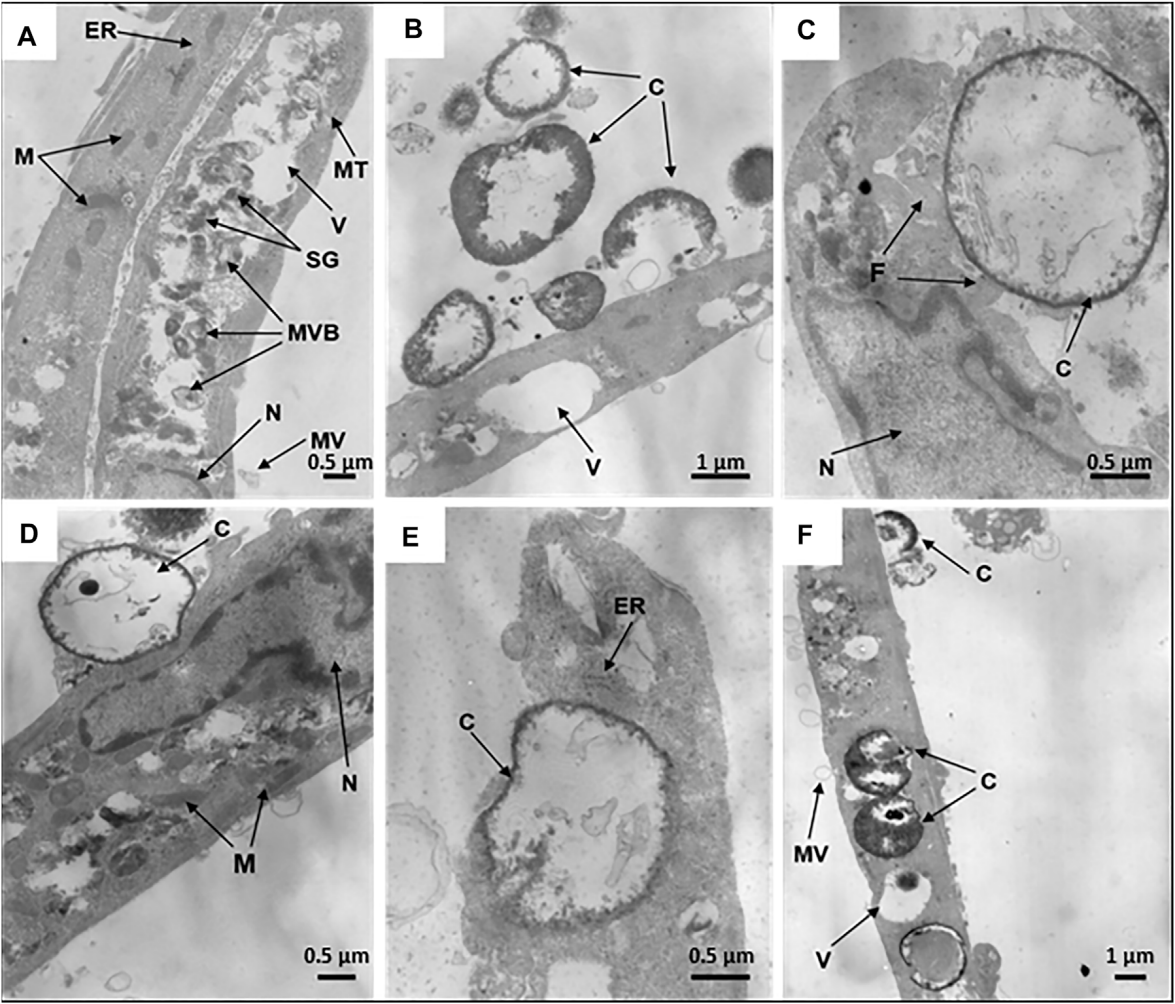
Internalization of synthetic microcapsules by hMSCs. Representative transmission electron micrographs of native hMSCs in the absence of microcapsules **(A)** and stepwise internalization of microcapsules 24 h post-phagocytosis at a 1:10 cell/microcapsule ratio. **(B)** Primary contacts of cells with microcapsules. **(C,D)** Capturing of microcapsules by filopodia or cells bodies, respectively. **(E)** Dipping of microcapsules into the cell cytoplasm. **(F)** Microcapsules inside a cell. Designations: C, microcapsules; N, nucleus; F, filopodia; ER, endoplasmic reticulum; V, vacuoles; M, mitochondria; SG, secretory granules; MT, microtubules; MVB, multivesicular bodies; MV, microvesicles.

The internalization efficiency and potential cytotoxicity are key parameters that need to be evaluated for further application of microcapsules in the functionalization of hMSCs. On transmission electron micrographs, control untreated hMSCs display an elongated shape with average linear dimensions of 16.9 ± 7.0 μm long axis and 2.7 ± 1.1 μm short axis. Membrane-derived micro vesicles were rarely observed on or near the cell surface. The cells display agranular cytoplasm containing numerous mitochondria with well-defined cristae, endoplasmic reticulum, lipid droplets, vacuoles of various size, and large nuclei, with a wrinkled surface, invaginations, peripheral heterochromatin, and nucleoli. In the cell bodies, dense secretory granules and multivesicular bodies are also observed (Figure 4A).

For investigation of the stepwise internalization of microcapsules within a cell, we used a 1:10 cell/microcapsule ratio at 24 h post-phagocytosis. We observed round, elongated, and “half-mooned” microcapsules both with intact or damaged membranes, either empty or filled with electron dense matter. First, one or more microcapsules became attached to a cell (Figure 4B), and then the cell formed “cavities” or invaginations by curving the cell membrane to internalize the capsules (Figures 4C,D). Alternatively, the surface-attached microcapsules were surrounded by filopodia (Figure 4D), following envelopment by the cytoplasm (Figure 4F). Finally, an hMSC could incorporate in the cytoplasm several microcapsules at a time (Supplementary Figure S2).

### Microcapsule’s Internalization Induces Dose- and Time-Dependent Ultrastructural Alterations of hMSCs

**FIGURE 5:**
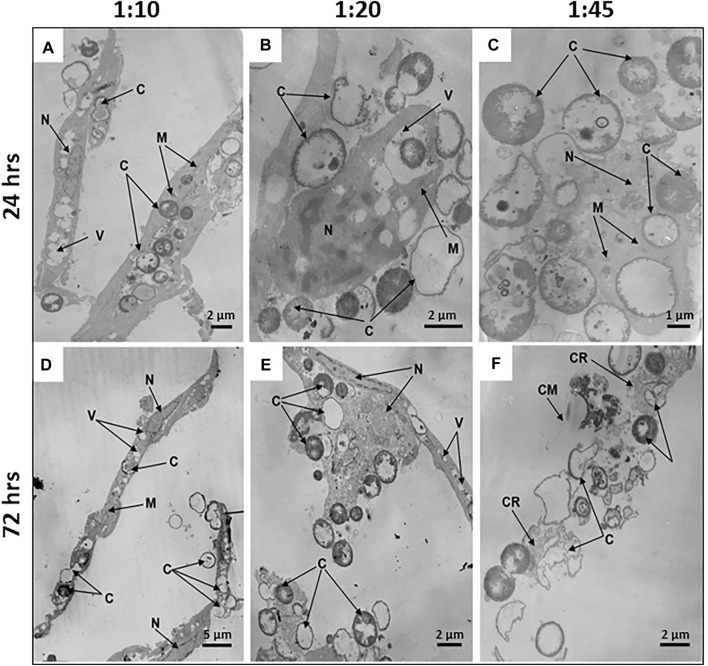
Microcapsule internalization induces dose- and time-dependent ultrastructural alterations of hMSCs. Representative transmission electron micrographs of hMSCs with microcapsules at 24 and 72 h post-phagocytosis at various cell/microcapsule ratios (1:10, 1:20, and 1:45). At 24 h post-phagocytosis hMSCs displaying **(A)** spindle-like shape at a 1:10 cell/microcapsules ratio, **(B)** irregular shape at a 1:20 ratio, or **(C)** significantly disrupted cells at a 1:45 ratio. The microcapsules were located both inside and outside the cells **(A–C)**. At 72 h post-phagocytosis hMSCs displaying **(D)** spindle-like shape, **(E)** irregular shape, or **(F)** were damaged. Designations: C, microcapsules; N, nucleus; V, vacuole; M, mitochondria; M, mitochondrium; CR, residual cytoplasm; CR, cytoplasmic remnants; CM, cell membrane.

To study time-dependent ultrastructural alterations of hMSCs induced by microcapsule internalization, cells were cultured for 24, 48, or 72 h following particle internalization at various doses. Transmission electron micrographs of each sample shows that microcapsules were located as extracellular, cell-attached, or intracellular particles. At 24 h post-cocultivation at a 1:10 cell/microcapsule ratio, virtually all hMSCs displayed a spindle-shaped morphology (indistinguishable from untreated control cells) with continuous intact cell membranes and clearly distinguishable organelles, including large nuclei with invaginations of the nuclear envelopes and elongated or round mitochondria with cristae (Figure 5A). At a 1:20 cell/microcapsule ratio, about 93% of the cells maintained the spindle-shaped morphology (as shown in Figure 5A), but there were also cells, comprising approximately 7% of the cell population, that had irregular shape with vacuoles, small mitochondria, and nuclei with a large amount of heterochromatin (Figure 5B). A substantial fraction of microcapsule-treated cells at 1:10 and 1:20 cell/microcapsule ratios contained 1–15 capsules per cell. At a 1:45 cell/microcapsule ratio, the hMSC population was quite heterogeneous and contained only about 65% spindle-shaped cells (as shown in Figure 5A), ∼20% of irregular-shaped cells (as shown in Figure 5B), and ∼15% of shapeless, significantly disrupted cells, characterized by degranulated cytoplasm with disrupted organelles, including a degraded nucleus and swollen mitochondria (Figure 5C); 24 h post-phagocytosis at the highest cell/microcapsule ratio, most cells, intact or damaged, contained 2–30 microcapsules.

48 h post-phagocytosis at all of the cell/microcapsule ratios studied, the cells were heterogeneous and formed the same three major subpopulations: normal-looking spindle-shaped cells, irregular-shaped, or largely damaged cells with disrupted organelles, some with residual cytoplasm. As the number of microcapsules per cell increased, the fraction of spindle-shaped cells decreased and the share of irregular-shaped and damaged cells increased, confirming cytotoxicity at higher microcapsule concentrations (data not shown).

72 h post-phagocytosis of hMSCs with microcapsules at a 1:10 cell/microcapsule ratio, we observed 88% of the cells with unaltered shape and ultrastructure similar to control cells (Figure 5D), but 12% of population were characterized by irregular-shaped cells with a damaged plasma membrane, degranulated cytoplasm, condensed nuclei, and swollen mitochondria with ruptured cristae. At 1:20 and 1:45 cell/microcapsule ratios, only a few hMSCs were spindle-shaped, while the number of irregular-shaped (Figure 5E) or disrupted hMSCs with only residual cytoplasm was much higher (Figure 5F). At 1:10 and 1:20 cell/microcapsule ratios, hMSCs contained 1–15 microcapsules per cell, whereas at a 1:45 cell/microcapsule ratio, they contained up to 40 microcapsules per cell.

### Increases in Microcapsule Loading Significantly Reduce hMSC Cellular Motility

**FIGURE 6:**
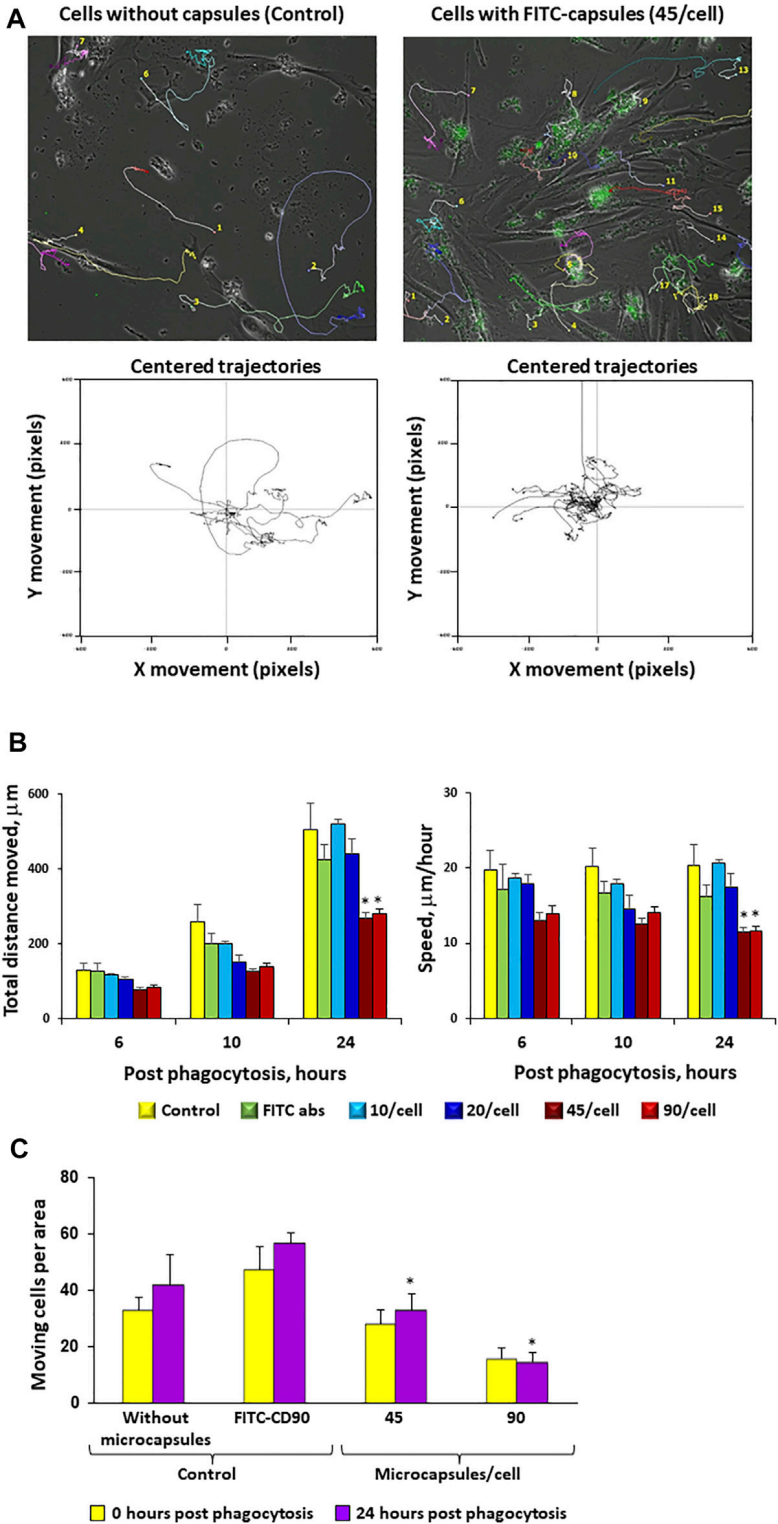
Increases in microcapsule loading significantly reduce cellular motility. Post-phagocytosis of microcapsules at ratios of 1:0 (control without capsules), 1:10, 1:20, 1:45, and 1:90 hMSCs were analyzed for cell motility for 24 h post-phagocytosis using the Cell-IQ v2MLF integrated platform for a continuous real-time phase-contrast imaging as described in the Methods. The cells were incubated with microcapsules for 2 h following filming during 24 h in the Cell-IQ system. Individual cells were marked by colored tags and traced for 24 h post-phagocytosis. Time-based traces of the images of cells loaded with 45 microcapsules/cell or 0/cell (control) over 24 h post-phagocytosis (**Panel A**, top panel). The lower panel shows the movement traces for individual cells with each trace being placed over the center of the window. Cells loaded with 45 microcapsules/cell exhibited shorter trajectory movement from center (**Panel A**, lower panel). From these data, cell motility can be assessed as total distance moved in an indicated time period (6, 10, or 24 h) or as average speed moved per hour **(Panel B)**. The **(Panel C)** total number of moving cells per area for each microcapsule ratio at indicated time post-phagocytosis. Cells incubated with CD90 fluorescent antibody were used as a control. These data demonstrate that increases in microcapsule loading significantly reduce cellular motility. *р < .05 *versus* the corresponding control at the same time point.

To evaluate whether microcapsules internalization can affect cell motility, cells loaded with microcapsules at various ratios were analyzed for their motility for 24 h post-phagocytosis as described in the Methods. Figure 6A demonstrates that cells loaded with microcapsules at a ratio of 1:45 exhibited shorter trajectory movement than control (0 microcapsule/cell). The total distance that the cell moved during all indicated time points and average speed per hour demonstrated that cells loaded with microcapsules moved shorter distance and with slower speed than cells without microcapsules or with FITC-labeled CD90 (Figures 6B,C). At 24 h post-phagocytosis, cells loaded with microcapsules at a ratio of 1:45 and 1:90 exhibited significantly shorter movement distance and slower speed than with control. There was no significant difference in speed per hour between cells loaded with microcapsules at a ratio of 1:10, 1:20, or control at all time points and at all ratios (Figure 6B). Thus, these data demonstrate that cell total movement distance and average speed per hour are microcapsule-loading dependent (Figures 6B,C). Figure 6C shows that the total number of moving cells per area is significantly reduced with an increase of microcapsule loading.

### Migration Activity of hMSCs Through 8 μm Pores Is Microcapsule Loading–Dependent

**FIGURE 7:**
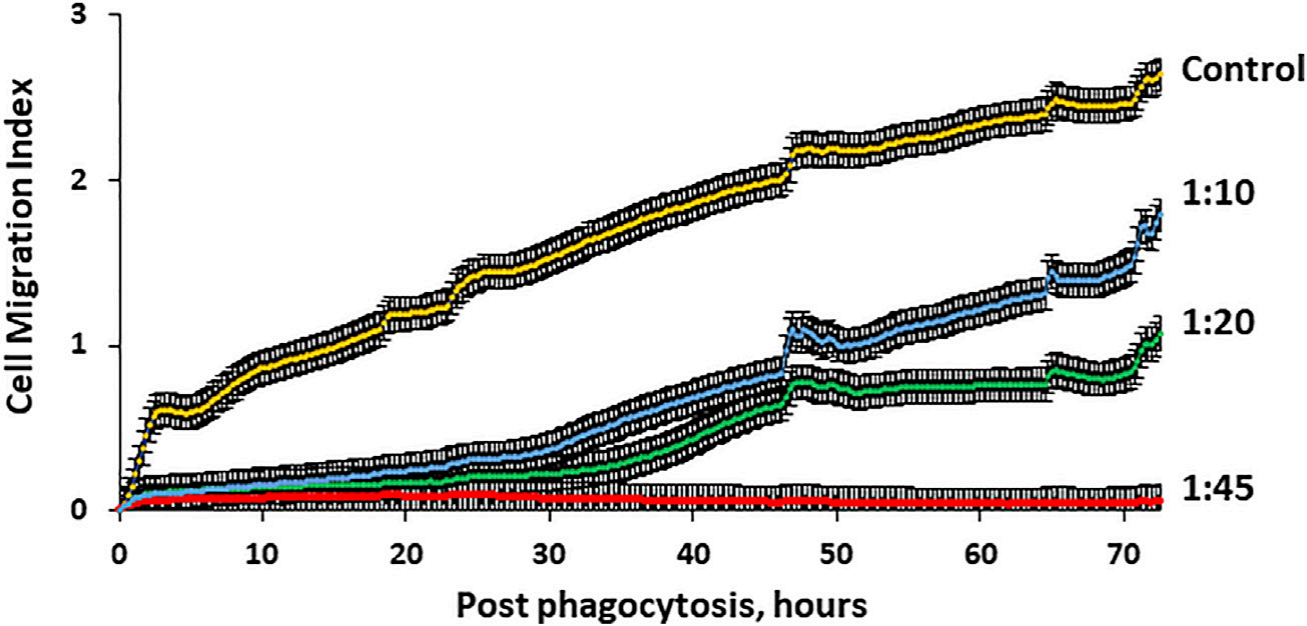
Migration activity of hMSCs through 8 µm pores is microcapsule loading–dependent. Human MSC migration through micropores was monitored with special 16-well CIM plates of the x CELLigence RTCA DP system as described in the Methods. The system allows real-time determination of the impedance dynamics at cell contact with gold electrodes and determination of the cell index correlating with the number and area of cells attached to the electrode. Cells (40,000) were placed in the upper chamber and allowed to migrate through 8 µm pores to the lower chamber which is 80% covered with gold electrodes. Four wells were used for each experimental group. Signals for the determination of the migration index were registered every 15 min for 72 h. These data demonstrate that cell migration activity through 8 µm pores was microcapsule loading–dependent and significantly reduced with higher microcapsule loading. *p* < .01 for cell/microcapsule ratios 1:10, 1:20, and 1:45 when compared with control according to the Wilcoxon test.

Since increases in microcapsule loading significantly reduce cellular motility, it is important to investigate whether microcapsule loading affects the ability of human MSCs to migrate through micropores. Thus, cells loaded with microcapsules at various ratios were placed in the upper chamber and allowed to migrate through 8 μm pores that imitate the blood vessel barrier. Cell migration activity through pores was monitored up to 72 h as described in the Methods. Figure 7 demonstrates that cells loaded with microcapsules at a ratio of 1:45 did not migrate through pores to the lower chamber at any monitored time. Cells loaded with microcapsules at lower ratios (1:10 and 1:20) migrated through pores but only at an insignificant rate during the first 30 h post-phagocytosis. However, after 30 h, the migration activity of cells with 1:10 and 1:20 microcapsules/cell increased significantly and continued to increase over time up to 72 h. Cell with microcapsules at a ratio of 1:10 demonstrated significantly higher migration index that those with a ratio of 1:20 but still significantly lower than control. These data demonstrated that cells, loaded with microcapsules at 1:10 and 1:20 ratios, maintained their ability to migrate through pores up to 72 h post-phagocytosis.

## Discussion

Targeted delivery of drugs, especially proteins and gene therapy systems, to specific organs and tissues is one of the most important directions in translational and clinical research. Among various drug-targeting approaches, cell-based delivery systems offer some unique strengths and have achieved exciting preclinical and clinical results. Cell-based targeted delivery systems are a promising delivery strategy because of their low immunogenicity and cytotoxicity, specific tropism to injured tissue, prolonged circulation time, and extended half-lives (Timin et al., 2018b; de la Torre et al., 2020). The use of immune cells as drug carriers was quickly identified as one of the promising delivery systems. Various cell types have been reported as effective natural carriers, including neutrophils, leukocytes, phagocytes, red blood cells, platelets, B and T lymphocytes, natural killer cells, tumor cells, and stem cells (Sun et al., 2017).

The use of mesenchymal stem cells as a targeted-delivery carrier is promising due to their following properties: ease of isolation, expansion, maintenance of their phenotype, multilineage potential *in vitro* (Ullah et al., 2015), and natural homing capability to injury, inflammation, and tumor (Compte et al., 2009; Fliervoet and Mastrobattista, 2016; Wu et al., 2018). MSCs can also be used to regenerate disease-affected tissues and to combat cancer, including lung adenocarcinoma, glioblastoma, and leukemia (Bagó et al., 2017; Hu et al., 2018; Layek et al., 2018; Salehi et al., 2018). In addition, a significant amount of research has been done to engineer stem cells for targeted delivery of genetic materials. MSCs engineered with mRNA encoding IL-10 could rapidly localize to local inflamed sites and lead to a superior anti-inflammatory effect by secreting IL-10 (Levy et al., 2013). It has been shown that MSCs can serve as a carrier of drugs in either free form or encapsulated within a nanoparticle (Cao et al., 2014; Pacioni et al., 2015). For example, the combination of MSCs with mesoporous silica nanoparticles targeting orthotopic glioblastoma demonstrated a higher tumor uptake of cell-carried nanoparticles than nanoparticles alone (Huang et al., 2013). Mesenchymal stem cell–based therapies are currently used in over 250 ongoing clinical trials (clinicaltrials.gov) as a potential therapeutic to treat multiple inflammatory diseases (Singer and Caplan, 2011), autoimmune diseases, and cancer (Zhao et al., 2020).

Encapsulation of biomacromolecules such as mRNA, DNA, proteins, or enzymes into polymeric micro- and nano capsules is a rapidly expanding area of research that is attracting considerable attention. Various encapsulation technologies have been developed; among them, the recently developed approach based on the layer-by-layer adsorption of oppositely charged species onto colloid-sized core particles seems to be one of the most promising approach due to its intriguing simplicity and versatility in terms of multiple components to be encapsulated in one entity (Timin et al., 2018b; Navolokin et al., 2018; Kudryavtseva et al., 2021). Recently, it was demonstrated that polyelectrolyte core-shell nanoparticles encapsulated with mRNA encoding GFP were successfully transfected into T cells *in vitro* (Tarakanchikova et al., 2020), and encapsulated mRNA encoding luciferase was successfully transfected into HEK293H cells *in vitro* (Kakran et al., 2015). It has also been shown that hMSCs functionalized with submicrometric silica-coated polymer capsules encapsulating an antitumor drug were successfully delivered into the tumor site and released drug upon near-infrared laser irradiation (Muslimov et al., 2020).

There are a few reports that characterize intact morphology and ultrastructure of human MSCs isolated from the bone marrow, amnion, placenta, and blood vessels (Colter et al., 2001; Raimondo et al., 2006; Pasquinelli et al., 2007a; Pasquinelli et al., 2007b; Crisan et al., 2008). However, there are no studies that characterize the ultrastructural features of cells during and after internalization of synthetic microcapsules to find the optimal conditions for cell–microcapsule interactions without cell ultrastructural damage. We used transmission electron microscopy as a method that enabled us to analyze the effects of synthetic microcapsules on the overall cell morphology and ultrastructure of human MSCs. The ultrastructure of intact hMSCs observed in our study (Figure 4A) is consistent with a previously published report (Manea et al., 2014) which described the ultrastructure of cells prepared from human lipoaspirate. The morphology and ultrastructure of microcapsule-treated hMSCs depended directly on the cultured post-phagocytosis time and the amount of microcapsule loading per cell. MSCs impregnated with microcapsules at ratios greater than 1:20 demonstrated dramatic shape changes from spindle-like to irregular associated with the degradation of organelles and shrinkage of the cytoplasm, indicating a moderate but distinctive cytotoxic effect (Figures 5C,F). The structural changes observed in Figures 5C,F correspond with increasing nitrite-level production (Figure 2) and with previously published data that artificial microcapsule uptake was associated with impaired cell adhesion due to detrimental interactions of the capsules with the cellular cytoskeleton during the internalization process (Lepik et al., 2016). MSCs incubated with microcapsules at lower ratios (up to 20 capsules per cell) demonstrated no visible changes in their structure (Figures 5A,D). These observations are in correspondence with small alterations in cytokines/chemokines (Supplementary Table S1) and nitrite levels in the media (Figure 2) at 24 and 48 h post-phagocytosis, suggesting that MSCs maintained their viability (Figure 1). According to Toma et al., MSCs leave blood stream circulation and infiltrate into the perivascular space of tissue by 48–72 h post-injection (Toma et al., 2009). Therefore, we to do not investigate the longer time points than 72 h post-phagocytosis.

One of the key features of hMSC loaded by microcapsules is their ability to migrate toward injury (Timin et al., 2018a). Our data demonstrated that MSC movement distance, speed, and ability to migrate through pores are microcapsule loading–dependent (Figure 6 and Figure 7) and maintained at lower amounts of microcapsule loading. Taken together, in this study, we have determined the ratios of microcapsules and time of post-phagocytosis that have a negligible effect on cell structure integrity, motility, and migration activity.

## Conclusion

In this study, we have demonstrated that human MSCs under appropriate conditions can be successfully impregnated with synthetic microcapsules without damaging cell structural integrity and mobility. We also demonstrated that hMSCs loaded with microcapsules still retained their motility and ability to migrate through 8 µm pores. This study establishes the possibility of using human MSCs as a carrier for targeted drug delivery of microencapsulated drugs or active genetic material, such as mRNA.

## Data Availability

The original contributions presented in the study are included in the article/Supplementary Material; further inquiries can be directed to the corresponding author.

## Citation

Litvinova LS, Shupletsova VV, Khaziakhmatova OG, Daminova AG, Kudryavtseva VL, Yurova KA, Malashchenko VV, Todosenko NM, Popova V, Litvinov RI, Korotkova EI, Sukhorukov GB, Gow AJ, Weissman D, Atochina-Vasserman EN, and Khlusov IA (2022) Human Mesenchymal Stem Cells as a Carrier for a Cell-Mediated Drug Delivery. Front. Bioeng. Biotechnol. 10:796111. doi: 10.3389/fbioe.2022.796111
