# Supplementary material for: Human Mesenchymal Stem Cells as a Carrier for a Cell-Mediated Drug Delivery
Source: Front Bioeng Biotechnol. 2022 Feb 24;10:796111. doi: 10.3389/fbioe.2022.796111 (PMC8909129; doi:10.3389/fbioe.2022.796111)
Supplement: Supplementary file 1 [file DataSheet1.PDF]

Supplementary Material

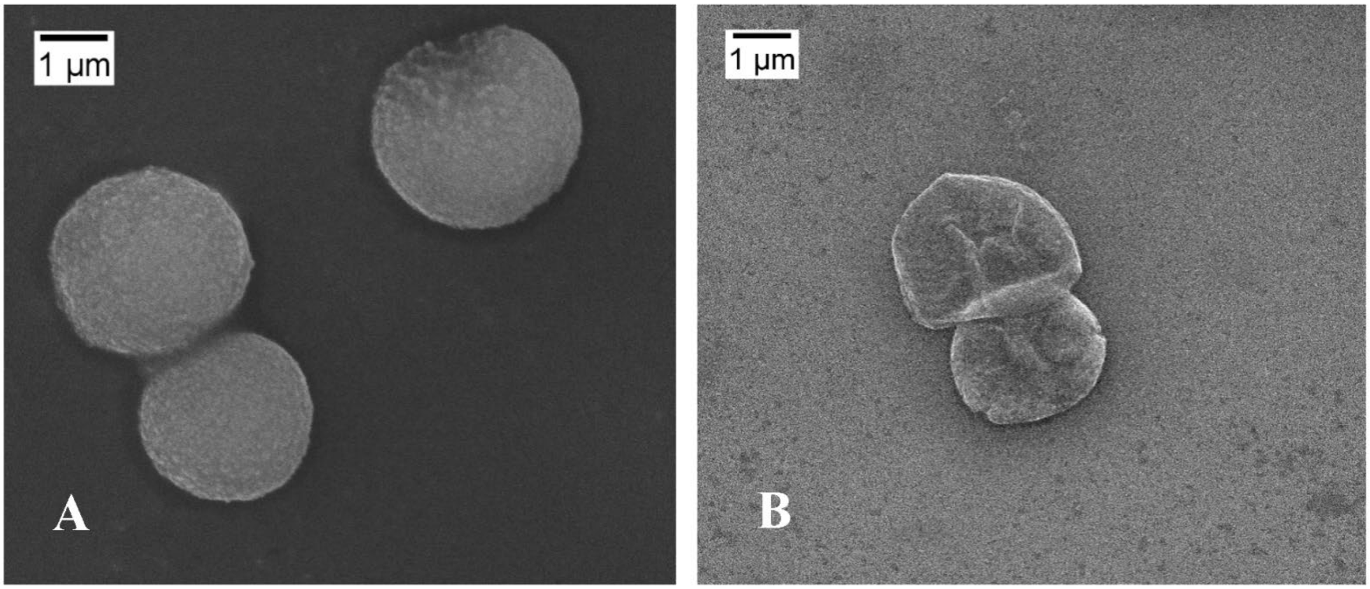

Supplementary Figure 1. Characterization of synthetized microcapsules.
Representative scanning electron microscope (SEM) images of a vaterite CaCO3 particles **(A)** and prepared polyelectrolyte capsules **(B).**

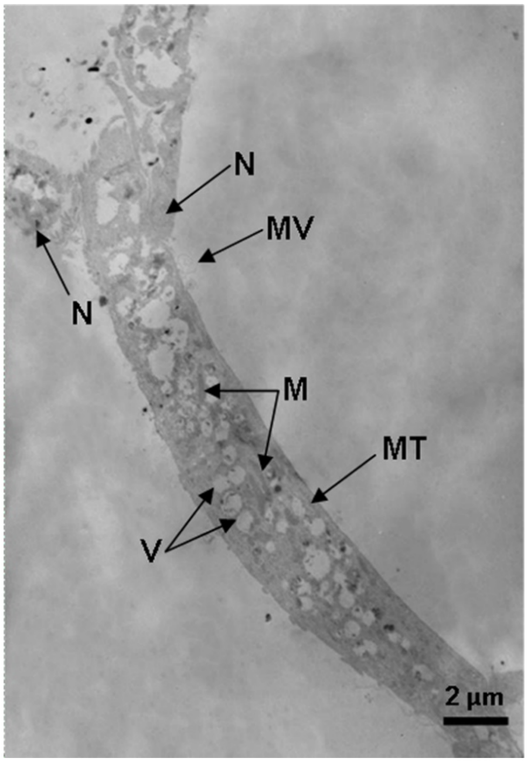

Supplementary Figure 2. Representative transmission electron micrographs of hMSCs in the absence of microcapsules at 72 hours. Designations: N - nucleus; V - vacuole; M – mitochondria; MT - microtubules; MV – microvesicles.

	24 hours post phagocytosis				48 hours post phagocytes			
**capsule/cell**	**0**	**5**	**20**	**45**	**0**	**5**	**20**	**45**
**IL-1α**	0.01 (0-0.88)	0 (0-0.77)	0 (0-0.64)	0 (0-0.46)	0	0 (0-1.04)	0 (0-1.65)	0 (0-0.23)
**IL-2rα**	4.38 (3.29-46)	6.50 (5.11-8.48)	4.43 (3.92-4.9)	5.40 (4.9-5.93)	45.33 (33.61-49.36)	39.36 (38.04-46.96)	8.44* (6.71-12.68)	6.24* (4.9-6.26)
**IL-3**	0.01 (0-0.08)	0 (0-0.11)	3.92 (0-7.42)	1.17 (0.04-3.11)	1.35 (1.32-1.83)	2.13 (1.29-3.19)	0*	20.58* (20.09-21.93)
**IL-12p40**	0.01 (0-3.43)	0 (0-8.91)	0 (0-21.55)	0 (0-8.91)	0 (0-16.41)	0.01 (0-24.83)	0 (0-18.15)	0 (0-37.29)
**IL-16**	10.91 (9.3-14.4)	18.4 (12.43-21.12)	15.3 (9.05-16.9)	12.52 (9.05-14.32)	36.76 (36.4-42.38)	46.87* (43.75-47.8)	13.35* (7.42-13.93)	19.35* (19.32-20.11)
**IL-18**	0.01 (0-0.01)	0 (0-0.41)	0 (0-0.05)	0	3.22 (2.75-4.53)	2.84 (1.2-4.35)	0.1* (0-0.15)	0.29* (0-0.93)
**CTACK**	0.98 (0.19-1.21)	2.22 (2.11-3.11)	5.32 (2.84-8.6)	3.32 (2.43-4.78)	32.09 (24.21-43.92)	31.79 (25.83-61.22)	5.4* (3.87-7.4)	2.81* (2.14-6.99)
**GROα**	9.43 (5.32-11.3)	31.86 (30.22-3.45)	62.3 (5.83-64.07)	82.34 (74.3-92.3)	22.70 (19.43-33.72)	47.54 (33.4-48.5)	83.23* (75.18-100.4)	193.4* (154.9-217.85)
**HGF**	3.21 (2.73-7.5)	5.42 (3.68-6.71)	5.28 (4.28-5.84)	2.77 (2.75-4.72)	30.06 (28.73-30.45)	27.44* (26.76-27.86)	20.52* (18.53-23.87)	11.74* (11.16-21.8)
**IFNα2**	0	0	0	0	0	0	0	0
**LIF**	6.9 (5.39-7.76)	9.43 (9.08-9.92)	9.83 (8.39-10.14)	9.98 (9.92-10.22)	12.61 (11.21-16.85)	12.33 (10.32-15.58)	13.2 (12.32-14.92)	14.13 (12.35-14.2)
**MCP-3**	13.12 (12.39-15.55)	16.35 (14.09-22.38)	20.06 (17.05-23.46)	16.69 (15.56-19.72)	65.55 (42.53-76.1)	58.49 (54.72-66.99)	47.9* (38.67-49.49)	27.7* (26.72-27.9)
**M-CSF**	3.43 (3.43-4.31)	5.32 (4.39-5.52)	4.92 (4.06-5.15)	5.40 (4.11-6.21)	6.43 (5.15-8.03)	8.03 (6.51-8.65)	4.74* (3.9-6.43)	4.4* (3.28-5.84)
**MIF**	98.21 (89.21-112.32)	119.4 (111.28-127.55)	36.9 (26.43-38.19)	28.7 (26.7-43.72)	31.34 (25.89-52)	78.32* (69.2-98.34)	110.3* (98.32-140.3)	175.4* (159.3-189.4)
**MIG**	2.1 (1.39-2.39)	5.43 (3.4-6.4)	5.42 (3.78-6.43)	13.43 (12.8-14.3)	14.2 (13.43-14.32)	18.6* (17.86-20.32)	8.6* (5.43-9.61)	6.11* (4.33-7.04)
**b-NGF**	2.39 (1.98-2.91)	2.34 (1.99-2.45)	2.45 (2.38-2.98)	3.62 (3.21-4.39)	5.11 (4.29-6.53)	4.93 (4.07-6.02)	6.43 (5.39-7.51)	2.03* (1.98-2.52)
**SCF**	0 (0-0.29)	4.46 (0-4.64)	3.55 (0-4.54)	4.76 (0-4.97)	6.59 (0-7.75)	7.67 (0-8.18)	5.84 (0-6.93)	5.58 (0-5.79)
**SCGF-b**	1123 (0-1329)	1372 (0-1637)	1241 (0-1432)	778 (0-874)	510.5 (0-622.4)	544.9 (0-721.32)	700.5 (0-794.66)	1287 (0-1298)
**SDF-1α**	21.32	29.4	27.82	31.71	60.95	67.82*	68.65*	70.37*

	(0.01-23.2)	(0-38.43)	(0-29.53)	(0-33.12)	(59.4-63.65)	(67.43-71.13)	(67.82-71.32)	(65.54-70.37)
**TNF-1b**	0<br>(0-0.39)	0.6<br>(0-0.83)	0.38<br>(0-0.6)	0.3<br>(0-0.6)	0.72<br>(0-1.19)	0.76<br>(0-1.01)	0.23<br>(0-0.45)	0.4<br>(0-0.95)
**TRAIL**	2.11<br>(1.39-2.11)	3.37<br>(2.98-3.54)	3.28<br>(2.48-3.37)	2.81<br>(2.37-3.29)	3.59<br>(3.39-5.37)	5.32<br>(4.7-5.43)	3.04<br>(2.29-3.11)	3.59<br>(2.46-3.7)

Supplementary Table 1. Secretory activity (pg/mL) of hMSCs after phagocytosis of microcapsules. Cell culture media were collected at 24 and 48 hours post phagocytosis with various ratios of microcapsules. Collected media were centrifuged at 500 g for 10 min and supernatants were analyzed by fluorescence flow cytometry by measuring the spontaneous and microcapsule-induced secretion of the following human cytokines and chemokines: LIF, SCF, SDF-1a, SCGF-β, M-CSF, MCP-3, MIF, MIG, TRAIL, Gro-α; IL-1a, IL-2ra, IL-3, IL-12 (p40), IL-16, IL-18, HGF, TNF-β, β-NGF, IFN-α2, and CTACK. Fluorescence flow cytometry was conducted with monoclonal antibodies according to the manufacturer's instructions for the cytokine assay system (Bio-Plex Pro Human Cytokine Group II 21-Plex Panel, Bio-Rad, Hercules, CA, USA) using an automated processing system (Bio-Plex Protein Assay System, Bio-Rad, Hercules, CA, USA). The concentration of each cytokine was presented in pg/ml, n=3-9 per group. The data are presented as Me (Q1-Q3). * statistically significant differences (<0.05) from control (0 microcapsules per cell; 1:0 ratio) according to Mann-Whitney U-test
